# Self-management training vs. neurofeedback interventions for attention deficit hyperactivity disorder: Results of a randomized controlled treatment study

**DOI:** 10.3389/fpsyt.2022.969351

**Published:** 2022-08-18

**Authors:** Ann-Kathrin Korfmacher, Oliver Hirsch, Mira-Lynn Chavanon, Björn Albrecht, Hanna Christiansen

**Affiliations:** ^1^Clinical Child and Adolescent Psychology, Department of Psychology, Philipps-University Marburg, Marburg, Germany; ^2^Department of Psychology, FOM University of Applied Sciences, Siegen, Germany

**Keywords:** children, attention deficit hyperactivity disorder (ADHD), neurofeedback training, self-management training, randomized controlled trial, neuropsychological test, quality of life, self-concept

## Abstract

**Objectives:**

Neurofeedback (NF) and self-management training (SMT) may be viable treatment options for patients with attention deficit hyperactivity disorder (ADHD) if they alleviate core symptoms, enhance the patients’ self-concept and improve their quality of life (QoL). Aim of the current study is evaluating both interventions accordingly and to test whether specific improvements in core symptoms lead to more general improvements in self-concept and QoL.

**Methods:**

In a psychotherapeutic outpatient clinic in Germany, a total of *N* = 139 children with ADHD were screened for eligibility, of which 111 fulfilled inclusion criteria and participated in the study in accordance with the CONSORT 2010 statement. These were randomly assigned to NF vs. SMT interventions. Changes from pre- to post-intervention in core ADHD symptoms relying on parent and teacher reports (CONNERS 3) and objective tests (Qb-Test) as well as self-concept (interview with the children) and QoL assessments (using the KINDL-R self-report) were compared between patients receiving NF or SMT.

**Results:**

Significant improvements in ADHD symptoms were achieved similarly in both treatment groups, whilst QoL and self-concept improved after SMT only.

**Conclusion:**

This treatment study provides further evidence that SMT and NF may reduce core symptoms, but SMT may also improve patients’ self-concept and QoL and may thus in its current form be the favorable treatment option in naturalistic settings. However, several limitations of the current study implicate that further research is required before definitive conclusions and recommendations for clinical practice can be given.

**Clinical trial registration:**

[www.clinicaltrials.gov], identifier [NCT01879644].

## Introduction

Both quality of life (QoL) and self-concept are central intrinsic aspects of health and well-being. The World Health Organization [(1), p. 1,450] defines QoL as “the individual’s perception of their position in life, in the context of culture and value systems in which they live, and in relation to their goals, expectations, standards and concerns.” This definition envisions QoL as a subjective evaluation embedded in a cultural, social, and environmental context, which should be realized by the individual and not by health professionals or family members. Self-concept describes a system of thoughts and attitudes about oneself (2) that becomes essential for mental health when someone encounters criticism and/or failure (3, 4).

Attention deficit hyperactivity disorder (ADHD) is a common neurodevelopmental disorder defined by the presence of Inattention, Hyperactivity, and Impulsivity across situations (5, 6). The disorder often persists into adulthood (7) and is associated with poorer academic, occupational, and social outcomes in the long-term (8). ADHD’s cross-situational and often persistent personal burden is reflected in patients’ psychosocial outcomes such as QOL and self-concept (9).

Children with ADHD and their parents typically report significantly lower QoL [(10); for meta-analyses see (11), (12)], although children rate their QoL higher than their parents do [see (10, 13)]. In addition, QoL is known to be impaired in several dimensions, especially in the psychosocial domains, and these subjective impairments are more pronounced the more severe the ADHD symptoms are and the older the affected person is [see review by Mulraney and Coghill (9); see meta-analysis by Lee et al. (12), López-Villalobos et al. (14), and Rocco et al. (15)].

More experience-dependent cognitive constructs such as self-concept may also be negatively affected by ADHD (16). For example, Safren et al. (17) model suggests that individuals with ADHD develop dysfunctional cognitions and beliefs as they grow older, facing new challenges and demands (e.g., in school and in their job), and underachieve or fail. These negative cognitions often take the form of negative appraisals that characterize depression (18) and contribute to lower levels of self-esteem and self-efficacy, and poor self-concept [e.g., (19, 20)]. These negative reciprocities between failures, frustrations and negative cognitions and beliefs may explain why impairments often persist even during a favorable course of the core symptoms [e.g., (21, 22); see overview in Wilens et al. (23)]. They may also account for the elevated risk of comorbid depressive or anxiety disorders across the life span [see (24)]. Although Safren et al. (17) model was developed primarily for (young) adults with ADHD, empirical studies support its validity also for youth: first, ADHD usually precedes depressive symptoms [see (25)]. Secondly, children and adolescents with ADHD report lower academic self-concept than their peers [e.g., (26–30)] and, thirdly, the deterioration of self-concept in children with ADHD depends on the ADHD subtype and global cognitive performance (31). Lastly, a recent longitudinal evaluation of the cognitive-behavioral model in a sample of young adults by Eddy et al. (32) also supported the main pathways proposed by Safren et al. (17) regarding college underachievement, negative self-concept, depressive symptoms, and functional impairment.

One important question that arises from all these findings is whether the treatment options for ADHD at hand also compensate for these negative, psychological consequences and functional impairments in a timely and sustainable manner [see also (9, 33)]. International guidelines advise that a comprehensive treatment program for ADHD should include psycho-education and may include behavioral therapy, parent training, and/or teacher-administered behavioral support, and, whenever necessary, medication (34, 35). Ideally, a favorable treatment should demonstrate that it can not only achieve a reduction in ADHD core symptoms over time, but also improves overall functioning (36), and interrupts the development of negative cognitive cascades, mapped, for example, in self-concept. Accordingly, in addition to symptom-based measures, functional impairment, QoL, and self-concept should also be considered in ADHD when evaluating treatments [e.g., see (13, 37)].

Following this call for patient-reported outcomes, QoL and psychosocial outcomes have recently come into the focus of research and clinical practice to prove whether a certain therapy improves both ADHD’s clinically relevant cardinal symptoms as well as the children’s general QoL.

As these efforts are still quite new, there are some randomized controlled trials (RCTs) of psychopharmacological treatments [e.g., (14, 38–40); see (41) or (9) for comprehensive reviews] and new treatment approaches [e.g., probiotic dietary intervention in Kumperscak et al. (42); high-intensity interval in Meßler et al. (43)] that have already considered QoL as an outcome. Although pharmacological interventions have yielded significantly improved QoL, the mean scores still remained significantly below the norm even after successful pharmacological treatment, indicating that medications alone may not achieve complete normalization [see overview in Mulraney and Coghill (9)].

There is evidence that cognitive behavioral therapy and psychoeducation may improve QoL in adults with ADHD [see (44) for a meta-analytic review]. There is also evidence that cognitive behavioral therapy may improve ADHD core symptoms [see meta-analyses of (45–49)], but only a few RCTs have comprehensively evaluated psychosocial outcomes such as QoL or self-concept in youth. For example, Larsen et al. (50) reported improved QoL after parent-training for preschoolers. In the study of Ferrin et al. (51), a program for parents of children/adolescents with ADHD including psychoeducation improved the pro-social domain. Another study by Kousha and Kakrodi (52) also evaluated psychoeducational training for mothers which raised the QoL of their children with ADHD. There is thus good evidence that parent training interventions and psychoeducation may improve QoL of children with ADHD (50–52). However, evaluations of interventions directly addressing self-management are currently rare; most of these studies dwell on ADHD core symptoms as primary outcomes, often ignoring QoL and self-concept (53–55). One of the few RCTs on neurofeedback (NF) identified impaired QoL in children with ADHD that failed to improve significantly after NF or biofeedback interventions (56), a finding in line with a less controlled study on NF of activity in the prefrontal cortex measured *via* near infrared spectroscopy (NIRS) (57). Another study, also on NF using NIRS that considers QoL is currently on its way [see (58) for the study protocol].

Accordingly, the present study aims to investigate the effects of two (neuro)cognitive/(neuro)behavioral interventions, namely neurofeedback (NF) and self-management training (SMT), on symptom- and function-based self-related cognitive measures.

The effectiveness of both SMT and NF to improve ADHD symptoms has been well studied [see e.g., (48, 59–61)]. Taking different approaches and applying various techniques, both NF and SMT are dedicated to promoting executive functions, in particular self-regulation skills. Aethiopathologically, these are supposed to represent one of the (core) deficits in the development of ADHD (62, 63), and studies have also indicated that executive deficits maintain their negative impact on QoL when controlling for ADHD symptom levels [e.g., (64)]. Accordingly, self-regulation training interventions appear promising for improving health and social outcomes beyond just reducing core symptoms (65). A popular German SMT intervention comprising two parts that proved effective in ADHD was introduced by Lauth and Schlottke (66). While the first part addresses selective attention, inhibitory control and basic self-regulation (e.g., stopping and checking), the second part teaches planning skills and self-instruction (66, 67).

Neurofeedback training of slow cortical potentials (SCPs) is another probably effective approach. It aims at first learning to control and self-regulate certain brain activity parameters (*via* real-time feedback and operant principles), and as the next step utilizing this ability (by transfer) to improve everyday life functioning [e.g., (68–72)]. The focus of the current study is on slow-frequency shifts in the electroencephalogram (EEG) that reflect the excitation of larger cortical cell assemblies (71, 73). These S can be shifted in negative polarity, indicating excitation in the underlying neuronal sources which may be associated with anticipatory or preparatory cognitive processes during task performance (74). Shifts with positive polarity seem to reflect an increase in the arousal threshold and consequently inhibition of activity in the corresponding functional networks (73, 75). In accordance with the cognitive-energetic model of ADHD (76), it has been consistently shown that children and adults with ADHD (and their first-degree relatives) exhibit less contingent negative variation (CNV), a functional negative slow potential evoked by processing task-relevant cues that require preparation for upcoming events (77–80) which may reflect at the neurophysiological level a dysfunctional regulation of energetic processing resources [e.g., (81)]. The neurobehavioral model of NF by Gevensleben et al. (82) summarizes established moderators of NF-training success [e.g., motivation to change, dealing with failure, and neurophysiological baseline profiles; see also (83)] and thus illustrates – once again – that factors such as self-concept and self-efficacy expectations can influence individual training success not only as outcome variables but also as moderators.

Overall, neurofeedback interventions are well evaluated. Recent meta-analyses showed (a) robust, small to medium immediate NF effects compared to non-active control especially regarding proximal ratings of Inattention (61, 84) that furthermore proved to be long-lasting (SMD_*pos*_t = 0.38; SMD_*FU*_ = 0.57), as well as (b) small but lasting effects on Hyperactivity-Impulsivity (SMD_*post*_ = 0.25; SMD_*FU*_ = 0.39) (84).

The aims of the present study are thus threefold:

1. To investigate whether the two (neuro-)behavioral therapy approaches of self-regulation, SMT and NF, exert a positive effect on QoL and self-concept in the affected children beyond ADHD core symptom improvement,
2. To investigate whether the two treatments exert a differential impact on symptoms, QoL, and self-concept,
3. And to predict which patient characteristics specifically predict favorable NT or SMT outcome regarding (a) ADHD core symptoms, (b) raising QoL, and (c) improving self-concept.

## Methods

### Participants

#### Inclusion and exclusion criteria

Participants were children referred to our psychotherapeutic outpatient clinic either by their parents, pediatricians, or psychiatrists for treatment of ADHD. Diagnoses were verified using a semi-structured diagnostic interview (K-SADS-PL) to examine the actual and past episodes of psychopathology in accordance with the DSM-III-R and DSM-IV criteria ((85); German adaptation (86)). The interview was conducted with the child’s parent(s) with a clinical rater assessing each symptom. To participate in the study, children had to meet the following inclusion criteria: age 7–12 years, fluency in German, current DSM-IV diagnosis of ADHD, IQ ≥80. Children with comorbid disorders were not excluded from the study, but the treatment of comorbid conditions was included in the treatment plan. Children taking stimulants were also not excluded from the study, but dose and potential changes were recorded.

Children with disorders or conditions that may mimic ADHD such as autism, brain disorders, epilepsy, hyperthyreosis, and any genetic or medical disorder associated with externalizing behavior were excluded from our sample [for a detailed description of the study protocol see (87)].

#### Final sample

**FIGURE 1:**
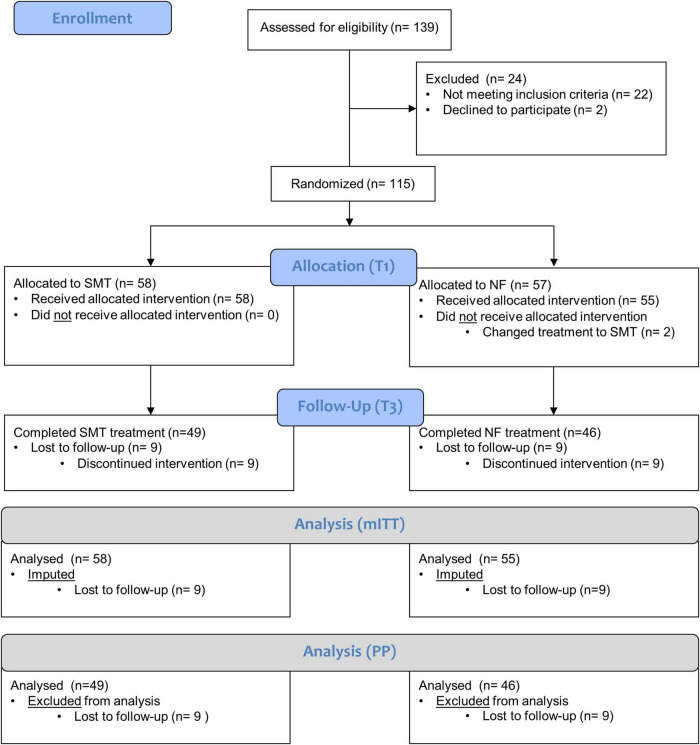
CONSORT flow-chart. For the current study, *n* = 139 children with ADHD were assessed for eligibility, of which *N* = 115 fulfilled inclusion criteria, gave final informed consent for participation and were randomized into groups receiving SMT (*n* = 58) or NF (*n* = 57) as the intention to treat (ITT) sample. Of these, *n* = 2 participants randomized into the NF group changed treatment to SMT and were excluded from modified mITT analyses. In the course of the intervention, *n* = 9 participants were lost in each group until follow-up (T3). Consequently, the mITT analyses comprises *n* = 58 children receiving SMT and *n* = 55 receiving NF whilst the per Protocol analyses comprises *n* = 49 children receiving SMT and *n* = 46 receiving NF that actually completed their interventions.

The study took place between 2011 and 2022 in the psychotherapeutic child and adolescent outpatient clinic at Philipps-University Marburg, Department of Clinical Child and Adolescent Psychology. A total of 139 children aged 6–11 years were screened for eligibility, of which *n* = 22 did not meet inclusion criteria and *n* = 2 refused participation (see Figure 1). The remaining sample (*N* = 115) was intended to treat, stratified for sex and medication and randomly assigned to SMT (*n* = 58) or NF (*n* = 57) interventions. As the study was planned as a naturalistic therapy study, *n* = 2 subjects assigned to NF had their treatment changed to SMT as recommended by the supervisor, so a total of *n* = 55 subjects started their NF intervention according to protocol and were included in the modified intention-to-treat (mITT) analyses. Parents of children were also randomized to one of two conditions: either parent training focusing on psychoeducation (PE) or PE involving additional social support (SU).

In total, *n* = 49 children completed SMT and *n* = 46 NF interventions and were included in the per protocol (PP) follow-up (T3) analyses (see Supplementary Material).

### Study design

This study compared SCP neurofeedback training (NF) with SMT (88). Both interventions took place very frequently, with three sessions per week over 3 months. There was a 1-week break after the 12th and the 24th session. Children were assessed before the training (T1), after 24 sessions (T2) and post-therapy (after 36 sessions; T3). Sessions 25–30 were devoted to potential comorbid problems and followed by an additional 6 highly frequent sessions (3/week) with either NF or SMT. Furthermore, three booster sessions were scheduled 6 months after end of therapy to activate the strategies learned. All children came in for follow-up assessments at 6 (T4) and 12 months (T5) after therapy. For details on study design and procedure, please refer to the study protocol (87). The current study analyzes data from T1 and T3.

This RCT study was examined and approved by the local ethics review board of the Department of Psychology of Philipps-University Marburg (AZ: 2010-04) in accordance with the CONSORT 2010 statement (89) and is registered as NCT01879644 (87).

### Outcome measures

In this study, we focused on the primary and secondary outcome measures, as described in the study protocol (87). As the **primary outcome**, these include ADHD symptoms assessed *via* the

• **Conners-3** scales for parents and teachers that assess the three core symptoms Inattention, Hyperactivity/Impulsivity, as well as executive functioning, learning problems, defiance/aggression, peer- and family relationships, oppositional defiant disorder and conduct disorder rated on a 4-point Likert scale (90, 91). The psychometric properties of the Conners-3 German version (92) have been proven to be satisfactory in our sample (SMT and NF separately) with internal consistency ranging between 0.742 to 0.944 at T1 and T3 (see Supplementary Tables 7a,8a).

**Secondary outcome** measures are

• **Qb-Test**. The Quantified behavior Test (Qb-Test) is a continuous performance test (CPT) with a combined registration of motor activity that quantifies ADHD core symptoms (93). The Qb-Test was approved by the Food and Drug Administration (FDA) in 2012 and exhibited adequate psychometric quality in a German validity study with Cronbach’s alpha between 0.60 and 0.95, and established three factor structure (94). In our sample (SMT and NF separately) internal consistency ranged between 0.569 and 0.968 at T1 and T3 (see Supplementary Tables 7b, 8b).
• **KINDL-R**. This self-report questionnaire for assessing health-related QoL in children and adolescents comprises in its revision 24 items rated on a 5-point Likert-scale that reflects well-being in different areas of life; dependent variables were the mean scores (95). The scales’ internal consistency ranged between 0.213 and 0.857 for our study’s samples (SMT and NF separately) at T1 and T3 (see Supplementary Tables 7b, 8b).
• **Self-concept interview** by Schöning et al. (30) differentiates the children’s self-concept regarding family, peers, school, and body. The categories are perceived QoL, self-worth, and social interaction that are rated on a 5-point Likert-scale; internal consistency reached between 0.449 and 0.870 in our study (T1 and T3 separately, see Supplementary Tables 7b, 8b).

### Statistical methods

As the first step, we checked the randomization regarding age, IQ, ADHD subtype, and the primary and secondary outcome measures at T1 in general linear models (GLMs) with the between-subject factor treatment (SMT vs. NF). Treatment outcome was tested in mITT and per-protocol (PP) analyses regarding change(Δ)-scores (T3 − T1) using GLMs with the between-subject factor treatment (SMT vs. NF); significant improvement (that is, whether the respective Δ change score differs in the direction of improvement from zero) was tested for each treatment with confidence intervals of *p* = 0.05. These analyses were conducted with the statistical software SPSS Statistics 26 (96).

In addition, we performed Multigroup Analyses (PLS-MGA) within the Partial Least Squares Structural Equation Modeling framework (PLS-SEM). PLS-SEM can be regarded as a variant of structural equation modeling which uses an ordinary least square regression-based method (OLS) in contrast to the maximum likelihood estimation procedure in covariance-based structural equation modeling. PLS-SEM is a variance-based approach and can handle small sample sizes, complex models, multiple variables, and makes almost no assumptions about the level of measurement of data and their distribution (97). This is an advantage over traditional methods like GLM. A further important advantage with PLS-SEM is that the method is robust to missing values (98). Traditional methods have to be observable variables, simple model structures, and error-free measured variables that rarely occur (98, 99). The PLS-SEM belongs to second-generation methods as a method to predict causality between variables and to explain variance in the dependent variables (98). Furthermore, in PLS-SEM model estimations statistical quality criteria of the applied measurement instruments are incorporated. In PLS-MGA, the respective pre–post-models were evaluated separately for SMT and NF groups. The path coefficients were regarded as a measure of change from T1 to T3 within each group. Standardized path coefficients of ≥0.20 can be regarded as relevant as an increase of 1 SD in a predictor results in an increase of 0.20 SD units in the outcome (98). PLS-MGA then calculates whether the difference between the path coefficients of the two groups is significantly different from 0. This was done using the Welch-test, which was regarded as a measure of difference between the two groups in the respective scale. To evaluate the measurement model, the outer (factor) loadings of variables on each of their latent constructs should be at least 0.708. On the other hand, variables with loadings ≥0.40 can also be included thanks to their contribution to content validity (100). The path weighting scheme was used for model estimation which standardizes the included variables. We bootstrapped with 5,000 samples to obtain tests of significance for path coefficients and outer loadings of variables forming latent constructs. The resulting *t*-values were then tested for significance. Factor loadings between the two groups were also tested for significant differences. We considered a *p*-value of ≤0.05 to be significant. A limitation of this method is that there is no global goodness-of-fit criterion.

The internal consistency of latent constructs with more than one manifest variable is measured by the composite reliability and Cronbach’s alpha scores. Values of 0.60 to 0.70 are acceptable in exploratory research, but in more advanced stages of research values of 0.70 to 0.90 should be achieved while values above 0.95 are undesirable, as they signal redundant items (98). The average variance extracted (AVE) shows the proportion of variance the constructs explain in their indicators. It is equivalent to the communality in factor analysis, and is regarded as a measure of convergent validity. A value of 0.50 should be reached which indicates that, on average, the construct explains half of the variance of its indicators.

The method of k-nearest neighbor (kNN) using R package VIM (101) was applied to replace missing or invalid values on several variables – regarding questionnaires on item level – ranging from *n* = 1 (1%) to *n* = 20 (20.4%, this was the case for teacher ratings, as indicated in the results section) missing values. The nearest neighbor approach is fully non-parametric and less prone to model misspecification. These calculations were done with the SmartPLS 3 program (102).

Prediction analyses were conducted separately in the SMT and NF treatment groups, with a regression analysis predicting the parent-rated ADHD Δ change with forward selection of regressors (p_(in)_ = 0.10) from the variables sex, age, IQ, parent-rated Conners DSM-Inattention, and DSM-Hyperactivity/Impulsivity presentation and medication for treating ADHD (psycho-stimulants or atomoxetine).

## Results

### Sample characteristics at baseline (T1) and power-analysis (modified intention-to-treat)

The available total sample intended to treat at T1 comprised 113 children (77% boys) with ADHD (34% inattentive, 11% predominantly hyperactive/impulsive, and 55% combined type) with a mean age of nine years and 1 month (range 7;0 to 11;8 years), and an estimated mean WISC-IQ of 104 (range 81–138). The sample size enabled us to detect at least small to medium sized within-subject Δ change scores (df > 54, *d* < 0.38; that is changes in outcome parameters following treatments) and medium-sized between-subjects effects [df = 111, *d* = 0.53; that is, differences between groups receiving SMT vs. NF, with a power of 1-β = 0.08 and significance level of α = 0.05 (two-tailed)]. Considering that the difference between SMT and NF may not exceed *d* = 0.2 and significance level was set at conventional level of α = 0.05, the power we achieved was not larger than 1-β = 0.18 (and also considering trends with α = 0.10 would also increase the power only slightly toward 1-β = 0.28).

The samples receiving SMT and NF did not differ in these demographics (all *F*_(1,111)_ < 1, *p*s > 0.5, see Table 1), the proportion of ADHD presentations (*^χ^*^2^_(2)_ = 1.15, *p* = 0.56) and almost all primary and secondary outcome parameters (all *p*s > 0.05, part. η^2^ < 0.02), with the exception of trends for parent-rated Conners’ Inattention (*F*_(1,111)_ = 3.6, *p* = 0.06, part. η^2^ = 0.03, more difficulties in patients receiving SMT) and the self-concept interview regarding peers (*F*_(1,111)_ = 3.2, *p* = 0.08, part. η^2^ = 0.03, more positive self-concept in those receiving SMT).

**TABLE 1:** Sample characteristics at baseline (T1, mITT).

	Self-management training *n* = 58 mean (SD)	Neurofeedback *n* = 55 mean (SD)	
Age (in years)	9.0 (1.3)	9.2 (1.4)	*F*_(1,111)_ = 0.37, *p* = 0.54, part. η^2^ = <0.01
Estimated HAWIK-IQ	104 (10.7)	103 (13.2)	*F*_(1,111)_ = 0.30, *p* = 0.59, part. η^2^ = < 0.01
Sex (% males)	79%	76%	*^χ^*^2^_(1)_ = 0.14, *p* = 0.71
ADHD presentation^1^ (%)			*^χ^*^2^_(2)_ = 1.15, *p* = 0.56
Hyperactive/impulsive	12%	9%	
Inattentive	38%	31%	
Combined	50%	60%	
Medication (total)	23.6%	21.1%	X^2^_(1)_ = 0.10, *p* = 0.76
Psycho-stimulants	21.8%	18.2%	
Atomoxetine	1.8%	1.9%	
Primary outcome			
Conners parent-rated^2^			
ADHD-index	71 (6.5)	69 (7.2)	*F*_(1,111)_ = 1.3, *p* = 0.26, part. η^2^ = 0.01
Hyperactivity/impulsivity (DSM)	68 (10.1)	70 (9.5)	*F*_(1,111)_ = 0.4, *p* = 0.53, part. η^2^ < 0.01
Inattention (DSM)	71 (6.1)	68 (8.3)	*F*_(1,111)_ = 3.6, *p* = 0.06, part. η ^2^ = 0.03
Conners teacher-rated^3^			
ADHD-index	60 (8.6)	62 (6.8)	*F*_(1,111)_ = 1.9, *p* = 0.17, part. η^2^ = 0.02
Hyperactivity/impulsivity (DSM)	64 (6.4)	65 (5.6)	*F*_(1,111)_ = 0.4, *p* = 0.55, part. η^2^ < 0.01
Inattention (DSM)	62 (6.6)	62 (6.1)	*F*_(1,111)_ = 0.1, *p* = 0.78, part. η^2^ < 0.01
Secondary outcome			
Qb-Test^4^			
Hyperactivity	0.63 (1.2)	0.43 (1.2)	*F*_(1,111)_ = 0.8, *p* = 0.39, part. η^2^ = 0.01
Impulsivity	−0.36 (0.9)	−0.30 (0.8)	*F*_(1,111)_ = 0.1, *p* = 0.75, part. η^2^ < 0.01
Inattention	1.81 (1.2)	1.68 (1.1)	*F*_(1,111)_ = 0.4, *p* = 0.55, part. η^2^ < 0.01
KINDL^5^			
Total	3.8 (0.5)	3.7 (0.5)	*F*_(1,111)_ = 2.0, *p* = 0.16, part. η^2^ = 0.02
Self-esteem	3.3 (1.0)	3.2 (1.0)	*F*_(1,111)_ = 0.9, *p* = 0.35, part. η^2^ < 0.01
Family	4.0 (0.7)	3.8 (0.7)	*F*_(1,111)_ = 2.1, *p* = 0.15, part. η^2^ = 0.02
School	3.7 (0.9)	3.5 (0.9)	*F*_(1,111)_ = 0.9, *p* = 0.35, part. η^2^ < 0.01
Peers	3.9 (0.9)	3.7 (0.8)	*F*_(1,111)_ = 0.7, *p* = 0.39, part. η^2^ < 0.01
Self-concept interview^6^			
Family	2.8 (0.6)	2.8 (0.5)	*F*_(1,111)_ = 0.5, *p* = 0.50, part. η^2^ < 0.01
School	2.7 (0.6)	2.5 (0.7)	*F*_(1,111)_ = 1.7, *p* = 0.19, part. η^2^ = 0.02
Peers	3.0 (0.6)	2.8 (0.6)	*F*_(1,111)_ = 3.2, *p* = 0.08, part. η ^2^ = 0.03

^1^According to DSM-IV as verified with the K-SADS, concordant with DSM 5. ^2^Missing for n = 6 children receiving SMT (n = 2) or NF (n = 4), imputed. ^3^Missing for n = 10 children receiving SMT (n = 2) or NF (n = 8), imputed. ^4^Missing for n = 24 children receiving SMT (n = 12) or NF (n = 12), imputed. ^5^Missing for n = 11 children receiving SMT (n = 3) or NF (n = 8), imputed. ^6^Missing for n = 6 children receiving SMT (n = 1) or NF (n = 5), imputed.

### Treatment effects from baseline (T1) to follow-up (T3), mITT on imputed data

The modified ITT analyses comprise data from all participants who received the allocated treatment according to randomization; these are *n* = 58 children treated with SMT and *n* = 55 (two dropouts due to changed treatment) treated with NF. Treatment effects were assessed with Δ change scores, computed as simple differences between measures at T3 minus T1; missing data was imputed, as described above and indicated in Table 2.

**TABLE 2:** Change in main outcome criteria from baseline (T1) to post-treatment (T3, mITT).

	Self-management training *N* = 58 mean (SD)	Neurofeedback *n* = 55 mean (SD)	
Primary outcome			
Conners parent-rated			
Δ ADHD-index	−6.2* (7.8)	−4.9* (8.2)	*F*_(1,111)_ = 0.7, *p* = 0.41, part. η^2^ < 0.01
Δ Hyperactivity/impulsivity (DSM)	−6.4* (9.9)	−6.8* (9.6)	*F*_(1,111)_ = 0.9, *p* = 0.34, part. η^2^ < 0.01
Δ Inattention (DSM)	−5.2* (7.7)	−3.7* (8.3)	*F*_(1,111)_ = 0.0, *p* = 0.86, part. η^2^ < 0.01
Conners teacher-rated			
Δ ADHD-index	3.0 (11.3)	1.9 (7.9)	*F*_(1,111)_ = 0.3, *p* = 0.57, part. η^2^ < 0.01
Δ Hyperactivity/impulsivity (DSM)	−3.9* (10.3)	−2.6 (8.3)	*F*_(1,111)_ = 0.1, *p* = 0.80, part. η^2^ < 0.01
Δ Inattention (DSM)	0.5 (9.6)	1.0 (7.8)	*F*_(1,111)_ = 0.6, *p* = 0.46, part. η^2^ < 0.01
Secondary outcome			
Responder-rate^2^			
Qb-Test			
Δ Hyperactivity	−0.6* (1.4)	−0.3 (1.5)	*F*_(1,111)_ = 0.9, *p* = 0.34, part. η^2^ < 0.01
Δ Impulsivity	−0.5* (1.2)	−0.6* (0.9)	*F*_(1,111)_ = 0.2, *p* = 0.65, part. η^2^ < 0.01
Δ Inattention	−0.4* (1.3)	0.0 (1.1)	*F*_(1,111)_ = 3.5, *p* = 0.07, part. η ^2^ = 0.03
KINDL			
Δ Total	0.2* (0.6)	0.0 (0.5)	*F*_(1,111)_ = 4.2, *p* = 0.04, part. η ^2^ = 0.04
Δ Self-esteem	0.3* (1.2)	0.0 (1.0)	*F*_(1,111)_ = 1.6, *p* = 0.22, part. η^2^ = 0.01
**Δ** School	0.2 (0.9)	−0.2 (1.0)	*F*_(1,111)_ = 4.9, *p* = 0.03, part. η ^2^ = 0.04
Δ Family	0.2 (0.8)	0.1 (0.9)	*F*_(1,111)_ = 0.1, *p* = 0.81, part. η^2^ < 0.01
Δ Peers	0.1 (1.0)	−0.0 (1.1)	*F*_(1,111)_ = 0.6, *p* = 0.46, part. η^2^ < 0.01
Self-concept interview			
Δ School	0.2* (0.7)	0.0 (0.7)	*F*_(1,111)_ = 2.3, *p* = . 13, part. η^2^ = 0.02
Δ Family	0.1 (0.5)	0.1 (0.6)	*F*_(1,111)_ = 0.3, *p* = 0.62, part. η^2^ < 0.01
Δ Peers	0.1 (0.6)	−0.0 (0.7)	*F*_(1,111)_ = 1.2, *p* = 0.28, part. η^2^ = 0.01

*Significant improvements from T1 to T3 (p < 0.05, two-tailed).

**FIGURE 2:**
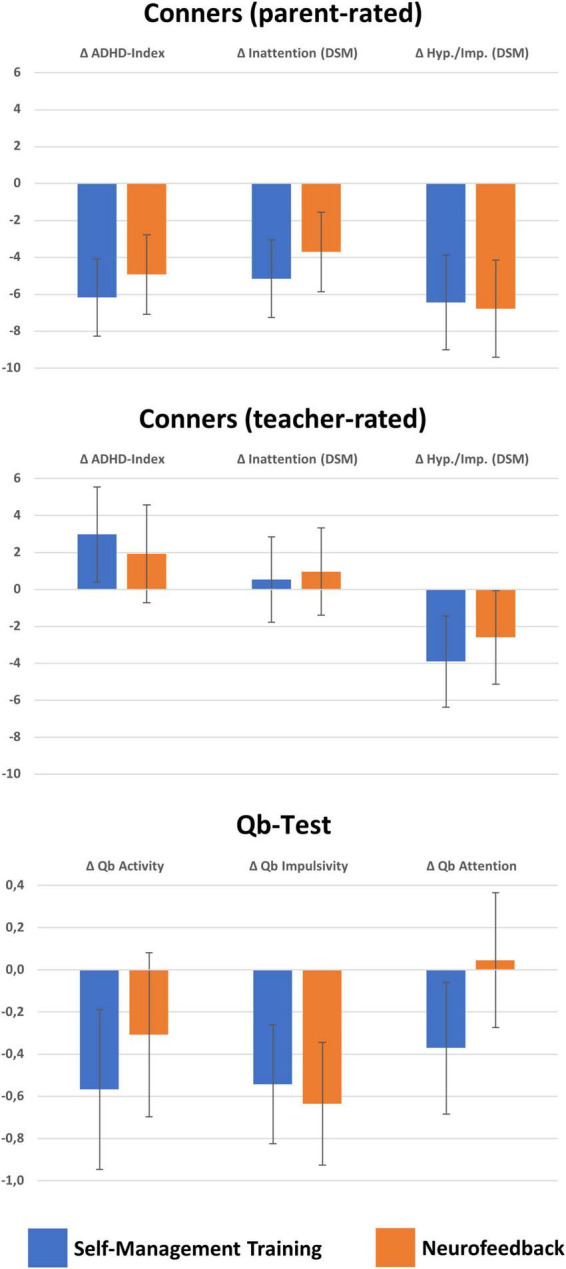
Changes in Conners parent- and teacher-rated and Qb-Test ADHD symptoms. This figure illustrates means and confidence intervals with *p* = 0.05 of ADND symptom changes from T1 to T3 from the modified intention to treat (mITT) analysis on imputed missing values. Significant improvements, that is, confidence intervals below zero as indicated by error bars, are in all of the parent-rated Conners scales (above), but in teacher ratings only in hyperactivity/impulsivity. The Qb-Test revealed improvements in particular for SMT for all ADHD symptom scores, but regarding NF only for Impulsivity. No significant differences in Δ change scores between treatments were detected except for Qb-Attention scores where SMT was superior to NF.

Regarding primary outcome measures, improved **Conners parent-rated** ADHD symptoms Δ ADHD-Index, Δ Hyperactivity/Impulsivity (DSM), and Δ Inattention (DSM) were eminent after both interventions (all *p* < 0.05, see Figure 2). However, potentially more blinded **Conners teacher ratings** indicated no benefit except for improved Hyperactivity/Impulsivity ratings after both treatments. Importantly, no significant differences between SMT and NF emerged (all *F*_(1,111)_ < 1, *p* > 0.34, part. η^2^ < 0.01).

**FIGURE 3:**
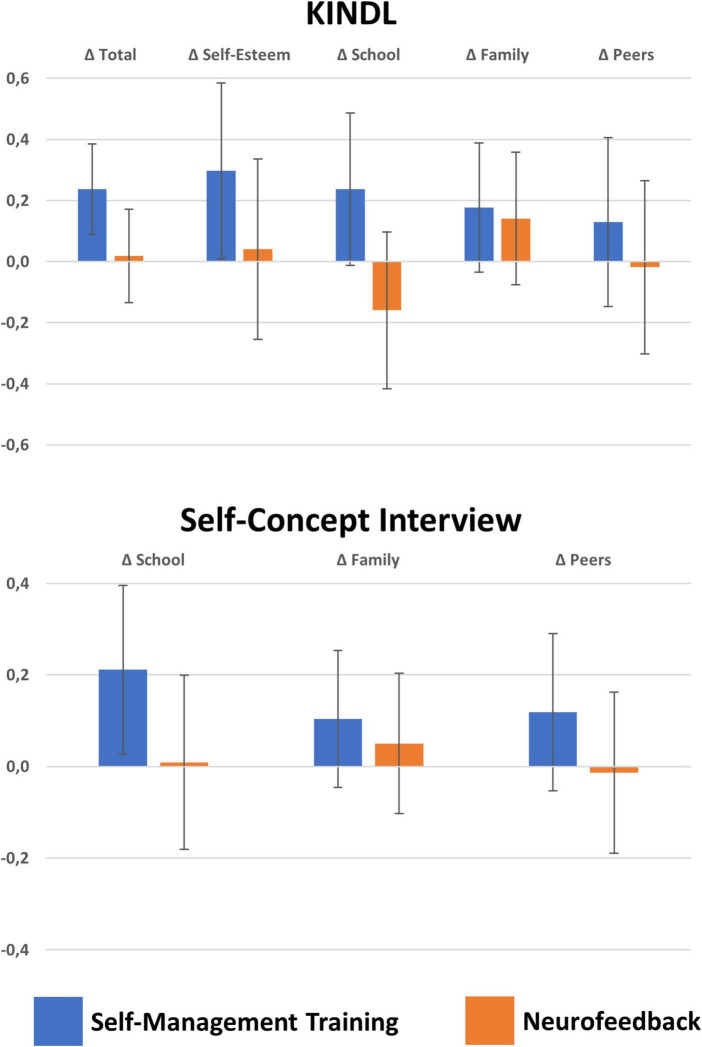
Changes in quality of life and self-concept. Means and confidence intervals with *p* = 0.05 of KINDL quality of life and self-concept scores. Significant improved quality of life was detected after SMT in self-esteem and marginally in school and family situations (*p* < 0.10), as well as self-concept at school whilst NF was not followed by any significant improvements. Direct comparisons between treatments revealed superiority of SMT over NF regarding quality of life in school.

Secondary outcome measures also revealed less difficulties after interventions. In particular, the **Qb-Test** quantified lower Δ Hyperactivity, Δ Impulsivity, and Δ Inattention after SMT whilst following NF only lower Δ Impulsivity was apparent. As a tendency, SMT was followed by stronger Δ Inattention improvements than NF (*F*_(1,111)_ = 6.5, *p* = 0.07, part. η^2^ = 0.03). QoL as assessed with the **KINDL** revealed improved in Total and in particular regarding Self-Esteem and at school for SMT, whilst no improvements in QoL were eminent after NF (see Figure 3); consequently SMT was superior to NF in these regards (Δ Total: *F*_(1,111)_ = 4.2, *p* = 0.04, part. η^2^ = 0.04, Δ Self-Esteem: *F*_(1,111)_ = 4.9, *p* = 0.03, part. η^2^ = 0.04). Moreover, the **self-concept interview** indicated also improvements at school following SMT, but no difference between treatments.

The respective outcomes of the PP analyses with and without imputations of missing values are given in Supplementary Tables 1,2.

### Multigroup analyses

There were no differences between the two groups in Conners’ subscales of parent and teacher ratings, as all differences between path coefficients were not significant. All but the path coefficient of the Conners’ Inattention Teacher ratings signaled significant changes from T1 to T3 within groups (for details refer to Table 3 and Supplementary Table 3 for the PP analyses). Factor loadings predominantly exceeded the minimum threshold of 0.40, and differences in the loadings between the two groups in Conners’ subscales were rarely significant (see Table 3).

**TABLE 3:** Original path coefficients of Conners subscales in parents and teachers in NF (*n* = 55) and SMT (*n* = 58) after imputing missing values with k-nearest neighbor (kNN) algorithm.

Scale	Path coefficients original (NF)	Path coefficients original (SMT)	*p*-value difference	Path coefficients bootstrap (NF)	Path coefficients bootstrap (SMT)
Conners hyperactivity parents	0.676	0.589	0.32	0.702 (*p* < 0.001)	0.634 (*p* < 0.001)
Conners inattention parents	0.569	0.466	0.63	0.605 (*p* < 0.001)	0.539 (*p* = 0.02)
Conners impulsivity parents	0.693	0.664	0.75	0.704 (*p* < 0.001)	0.670 (*p* < 0.001)
Conners hyperactivity teachers	0.684	0.671	0.90	0.701 (*p* < 0.001)	0.693 (*p* < 0.001)
Conners inattention teachers	0.366	0.499	0.65	0.400 (*p* = 0.20)	0.542 (*p* < 0.001)
Conners impulsivity teachers	0.644	0.631	0.91	0.655 (*p* < 0.001)	0.640 (*p* < 0.001)

Original path coefficients of Conners subscales in parents and teachers in neurofeedback (NF) and self-management (SMT) groups, the p-value of the Welch-test of differences and the mean path coefficients in both groups after 5,000 bootstrap samples and their significance values.

There were no differences between the two groups in the Qb-Test subscales, as all differences between path coefficients were not significant. All but the path coefficient of the Impulsivity subscale in the SMT group were significant, signaling significant changes from T1 to T3 within the groups. For details see Table 4 and Supplementary Table 4 for the PP analyses. Factor loadings of Qb-Test subscales predominantly exceeded the minimum threshold of 0.40 and did not differ between the two groups.

**TABLE 4:** Original path coefficients of Qb-Test subscales in NF (*n* = 55) and SMT (*n* = 58) after imputing missing values with k-nearest neighbor (kNN) algorithm.

Scale	Path coefficients original (NF)	Path coefficients original (SMT)	*p*-value difference	Path coefficients bootstrap (NF)	Path coefficients bootstrap (SMT)
Qb-Test hyperactivity	0.295	0.334	0.86	0.322 (*p* = 0.07)	0.350 (*p* = 0.023)
Qb-Test inattention	0.589	0.444	0.29	0.605 (*p* < 0.001)	0.491 (*p* < 0.001)
Qb-Test impulsivity	0.428	0.325	0.78	0.460 (*p* < 0.001)	0.132 (*p* = 0.35)

Based on Q values of Qb+ variables. Original path coefficients of Qb-Test subscales in neurofeedback (NF) and self-management (SMT) groups, the p-value of the Welch-test of differences and the mean path coefficients in both groups after 5,000 bootstrap samples and their significance values.

All KINDL path coefficients signaled significant changes from T1 to T3 within groups but no group differences (see Table 5 and Supplementary Table 5). Most of the KINDL subscales’ factor loadings exceeded the minimum threshold of 0.40 and revealed no significant differences between the two groups.

**TABLE 5:** Original path coefficients of KINDL subscales in NF (*n* = 55) and SMT (*n* = 58) after imputing of missing values with k-nearest neighbor (kNN) algorithm.

Scale	Path coefficients original (NF)	Path coefficients original (SMT)	*p*-value difference	Path coefficients bootstrap (NF)	Path coefficients bootstrap (SMT)
KINDL physical	0.422	0.399	0.92	0.483 (*p* = 0.005)	0.460 (*p* = 0.021)
KINDL self-esteem	0.586	0.369	0.22	0.611 (*p* < 0.001)	0.403 (*p* = 0.065)
KINDL family	0.491	0.388	0.39	0.550 (*p* < 0.001)	0.501 (*p* = 0.007)
KINDL peer	0.491	0.508	0.93	0.524 (*p* = 0.022)	0.561 (*p* < 0.001)
KINDL school	0.334	0.468	0.34	0.438 (*p* = 0.096)	0.525 (*p* = 0.001)
KINDL emotional	0.461	0.539	0.87	0.447 (*p* = 0.18)	0.477 (*p* = 0.11)

Original path coefficients of KINDL subscales in neurofeedback (NF) and self-management (SMT) groups, the p-value of the Welch-test of differences and the mean path coefficients in both groups after 5,000 bootstrap samples and their significance values.

There were no group differences in the self-concept interview subscales, as all differences between path coefficients were not significant (Table 6 and Supplementary Table 6). All path coefficients signaled significant changes from T1 to T3 within the groups. Most of the factor loadings exceeded the minimum threshold of 0.40, and the loadings between groups did not differ significantly in the self-concept interview subscales.

**TABLE 6:** Original path coefficients of self-concept interview subscales in NF (*n* = 55) and SMT (*n* = 58) after imputing missing values with k-nearest neighbor (kNN) algorithm.

Scale	Path coefficients original (NF)	Path coefficients original (SMT)	*p*-value difference	Path coefficients bootstrap (NF)	Path coefficients bootstrap (SMT)
SC body	0.483	0.632	0.16	0.551 (*p* < 0.001)	0.669 (*p* < 0.001)
SC family	0.584	0.636	0.55	0.670 (*p* < 0.001)	0.693 (*p* < 0.001)
SC peers	0.572	0.527	0.87	0.609 (*p* = 0.005)	0.576 (*p* = 0.005)
SC school	0.510	0.416	0.63	0.593 (*p* < 0.001)	0.534 (*p* = 0.013)

Original path coefficients of self-concept interview subscales in neurofeedback (NF) and self-management (SMT) groups, the p-value of the Welch-test of differences and the mean path coefficients in both groups after 5,000 bootstrap samples and their significance values.

### Prediction of treatment outcome

We analyzed the mITT sample regarding the primary outcome Conners parent-rated Δ ADHD-Index. Treatment outcome for those receiving the SMT PP is predicted by the regression model (*F*_(2,55)_ = 6.2, *p* < 0.01), including parent-rated DSM Inattention (standardized β = −0.35, *T*_(55)_ = −2.9, *p* < 0.01) and parent-rated DSM Hyperactivity/Impulsivity (standardized β = −0.28, *T*_(55)_ = −2.2, *p* = 0.03): patients with more difficulties improved more. NF outcome PP is predicted (*F*_(1,53)_ = 6.0, *p* = 0.02) by parent-rated Conners’ DSM-Inattention (standardized β = −0.32, *T*_(53)_ = −2.5, *p* = 0.02), and we noted a greater reduction in symptoms in patients with more Inattention difficulties. Interestingly, sex (both *p* > 0.32), age (both *p* > 0.11), IQ (both *p* > 0.77), and pharmacological treatment for ADHD (both *p* > 0.12) did not predict treatment outcome following SMT or NF.

Improved QoL in KINDL Total scale was only predicted as a tendency in those receiving SMT by parent-rated DSM Inattention (*F*_(1,56)_ = 3.5, *p* = 0.07, standardized β = 0.24, *T*_(55)_ = 1.9, *p* = 0.07); no prediction was significant in the NF group.

## Discussion

In the present study, we evaluated the effectiveness of two frequently conducted (neuro)behavioral therapies that dwell on self-regulation in children with ADHD regarding their beneficial impact on the core symptoms, QoL and self-concept. To this end, we chose a naturalistic setting to investigate (1) whether SMT and NF interventions lead to improvements in QoL and self-concept that go beyond reducing symptoms, (2) whether the two treatments have a differential impact on the QoL and self-concept, and (3) whether we would be able to predict for whom NT or SMT is especially beneficial in alleviating (a) ADHD core symptoms, (b) in QOL, and (c) in self-concept. We hypothesized that SMT and NF would significantly reduce core ADHD symptoms and improve QoL and self-concept (87).

The main results for the ADHD core symptoms showed on the one hand that particularly the proximal, but unblinded parent-rated Conners’ ADHD symptom scores decreased in both groups compared to baseline, while more distal and probably blinded teacher ratings revealed improved Hyperactivity/Impulsivity only. This discrepancy may also reflect true differences in reference and perception, as teacher rely on more structured situations in light of academic achievement (where hyperactivity/Impulsivity may be more disturbing and get more noticed), whilst parents rate their children in less structured situations at home [see e.g., (103)]. On the other hand, the objective Qb-Test showed improvements in all three ADHD core symptoms in SMT, but only for Impulsivity in children who underwent NF. Beyond these improvements in ADHD core symptoms, we detected some improved QoL, in particular in self-esteem and as trends, also at school and in family situations, following SMT but clearly not after NF. In addition, children with more Inattention difficulties improved more from SMT and NF. In contrast, patients’ sex, age, and IQ as well as medication for treating ADHD (in particular psychostimulants and with low frequency atomoxetine) showed no prediction capacity for treatment-outcome. We now discuss each of these findings in more detail before sketching some implications for future research and treatment for children with ADHD.

### Improvements in primary outcomes

The mandatory primary outcome of treatment for ADHD is that it must reduce the core symptoms. In the present study, we observed this effect in the Conners’ parent ratings of DSM Inattention and Hyperactivity/Impulsivity, and in ADHD Index ratings after both treatments. However, these promising findings may be rather unspecific, as parents are entirely aware of the treatments and may overrate treatment benefits, e.g., through placebo effects [see e.g., (104, 105)]. Such bias is also observable in more distal ratings such as those of teachers, though these may be less prominent in such ratings. However, both treatments studied here resulted in alleviated Hyperactivity/Impulsivity, but not Inattention. This discrepancy is quite typical (106), though Hyperactivity/Impulsivity is easier to observe and more challenging in structured situations than are Inattention symptoms (107). As such, Hyperactivity/Impulsivity ratings may cover the more obvious, more disruptive aspect of the difficulties associated with ADHD and may thus be especially sensitive for treatment outcome in the short-term.

In addition to behavioral ratings, we also assessed ADHD symptoms with the Qb-Test as a standardized, objective test for ADHD symptoms that may be largely unbiased (94, 108). It revealed, in line with the behavioral ratings by parents and teachers, also alleviated (Hyper-) Activity and Impulsivity after both SMT and NF, while SMT moreover also reduced the Attention difficulties that may be uncovered in teacher ratings. In direct comparison, both treatments led to similar improvements regarding Activity and Impulsivity, but SMT was superior to NF in improving attention.

The results of the PLS-MGA are generally in line with the GLMs tested above: on the one hand, improvements in primary and secondary outcome measures appeared following both interventions, but differences between SMT and NF were not significant. Besides general agreement, this stands in contrast to SMT’s superiority regarding improved Qb-Attention, Total QoL, and improved self-concept at school detected in the conventional analyses of GLMs. One may speculate whether the parametric analyses had more statistical power, and it is important to consider the violation of the statistical assumptions, though PLS-SEM considers the psychometric properties within the model and is robust against violation.

The prediction of treatment outcome in terms of lower ADHD behavioral problems showed that children with more Inattention difficulties achieved greater improvements after both treatments, while those with elevated Hyperactivity/Impulsivity problems were more effectively treated with SMT. This result is in line with the those of Krepel et al. (109), who found that higher Hyperactivity led to non-remitters in NF. Interestingly, neither patient’s age nor IQ predicted any treatment’s outcome; a somewhat surprising finding considering that self-regulation skills *per se* are supposed to improve as children grow up and seem to be better in those with higher cognitive ability. One may speculate whether such associations are perhaps not be prominent enough in our sample of relatively young patients within a limited age-range (7–12 years and a SD of about 1.3 years). In contrast, IQ was within normal range and spread largely (IQ of 81 till 138 with a total mean of 104 and SD of 12.0) and differences according to IQ would have been expected.

Somewhat surprisingly, but because of the quasi-experimental variation in our study difficult to interpret, is the lack of significant beneficial effects of medication on neither SMT nor NF outcomes. Superiority of combined psychopharmacological and behavioral treatment over each element administered alone was indicated by several RCTs, e.g., the MTA-Study in which medication with psychostimulants, behavioral treatment, a combination of both and standard community care were randomly assigned to a large sample of children with ADHD (110). In contrast, our study randomly assigned SMT and NF to children with ADHD, but not medication. Consequently, medication may in our sample be instrumental for those patients receiving it, and not instrumental for those not receiving it. This quasi-experimental confound makes it difficult to interpret our finding that medication status is no significant predictor of treatment outcome beyond the plain “phenomenological” conclusion that patients receiving medication may profit similarly from both intervention as patients that do not receive medication – we simply do not know what happens with treatment outcome if those patients receiving medication stop it and those who did not receive medication start it.

### Improvements in secondary outcomes

The study by Rocco et al. (15) demonstrated the importance that treatments should not only focus on symptom relief but also on improving QoL, as ADHD negatively affects QoL of children and their families.

There are some indicators that QoL as measured with the KINDL in Total and at school, as well as the self-concept in school situations improved particularly after treatment with SMT, but clearly not after NF. This is in line with Strehl et al. (56), who also detected no significant improvement in (Total) QoL after NF, although their patients showed impairments. As we regard improved QoL and self-concept as essential indicators of treatment success, this seems to be an important point supporting SMT, but these findings failed to be significant in our PLS-MGA analyses. One may can speculate whether the skills elaborated in SMT through school-like tasks may be more easily transferred into school-like situations than self-regulation of brain activity acquired by NF taking place foremost in the laboratory with limited transfer when not conducted properly. While elaborated transfer was not achieved in NF, this might have been the case for SMT, as the critical transfer is implicated by treatment context. Such considerations aside, our data’s PLS-MGA indicated improvements with both SMT and NF, with no differences between treatments, so it remains open for future investigations to test whether SMT procedures may indeed be superior to NF, and whether optimized transfer may improve the effectiveness of NF further.

### Implications for future research

Attention deficit hyperactivity disorder impacts many areas of everyday life including cognitive, academic, social, and occupational development, as well as the patient’s self-concept and QoL (5, 6, 111–114). Comprehensive treatments should therefore alleviate these psychosocial impairments, but they are often considered secondary (36) and not a core research target.

In addition, the NF training sessions need stimulating variation, as that can influence motivation (82). For this, children take different training objects (e.g., a unicorn, shark, airplane, and much more) at every NF session [see (87)]. Both groups’ dropout rates were the same at T3 in our study, which may indicate that the variety of tasks and of training resources served as well in the NF group as in SMT.

It is very important that the transfer to everyday life also be integrated within therapy, especially for NF, so that self-control of certain brain activity can be practiced in relevant settings such as school (69, 115). Transfer to different settings was integrated in both study protocols as children were given small laminated cards with either the training strategies (SMT) or the object trained with (NF), and were asked to take those cards to school and to place them somewhere visible. Further, in the weeks between the highly frequent therapy sessions, children and parents were explicitly instructed to practice with those cards to train their children’s mental strategy in different everyday-life situations [see (87)].

Psychosocial interventions have often implemented psychoeducational parenting training interventions to improve psychosocial outcomes like QoL (50–52). We include this aspect in our study with two different parent groups [psycho-education only (PE) and PE + social support (SU)]. This is important so that parents can support their children in the transfer to everyday life [see (87)].

### Limitations

Our RCT was implemented in a psychotherapeutic outpatient clinic with both treatments (SMT and NF) provided (a) by psychologists trained for the study, but not yet licensed trainers and (b) as a high-frequency intervention.

First, the KINDL-R and self-concept interview should be interpreted with caution due to their low internal consistency. In the KINDL-R, this is especially true for the emotional, school, and physical scales, and in the self-concept interview, this concerns in particular the Body scale. These scales consist only of four items, which may have made it difficult to achieve internal consistency within an acceptable range.

Another aspect is that such high-frequency therapy was challenging for families due to other responsibilities like work, caring for siblings, and other daily commitments, which also contributed to some families dropping out of the study. There were quite a few non-starters or drop-outs in this study, which influenced our sample size and hence statistical power. The available sample size enabled us to detect the expected medium-sized within-subject improvements following SMT and NF, but the expected rather small difference between these two probably effective treatments may remain uncovered. Consequently, our study may suffice for detecting treatment effects, but is clearly limited when it comes to a direct comparison between SMT and NF.

In planning this study, we focused on the high personal burden of children with ADHD (9), and we did not consider it ethically justifiable to include a control group with an ineffective treatment to control for unspecific or even spurious effects. Consequently, the current study suggests that both treatments are effective at the end, but as an important limitation we have very little to say regarding specific modes of action required before definitive conclusions can be drawn.

In addition, we have already discussed the problem associated with our study protocol’s non-blinded setting, as well as the need to involve parents in the therapy of children with ADHD (87). However, in such a treatment study, it is virtually impossible to blind participants, their relevant caregivers or therapists, as this would hamper the patients’ self-efficacy expectations. Further, as this was a naturalistic treatment study, analyzing whether NF might be a viable treatment alternative in an outpatient setting, blinding was not a primary goal.

## Conclusion

Despite the difficulties associated with a naturalistic clinical study in an outpatient setting, we were able to demonstrate that all in all, both NF and SMT treatments are potentially effective in helping children with ADHD to not just reduce their core symptoms, but also to raise their QoL and self-concept. In light of our investigation’s limitations, further research is required before definitive conclusions and recommendations for routine care can be given, but the present report illustrates that therapy guidelines should also consider psychosocial outcomes and predictors of effectiveness when evaluating treatments.

## Data availability statement

The raw data supporting the conclusions of this article will be made available by the authors, without undue reservation.

## Ethics statement

The studies involving human participants were reviewed and approved by the local ethics review board of the Department of Psychology at the Philipps-University Marburg. Written informed consent to participate in this study was provided by the participants’ legal guardian/next of kin.

## Author contributions

A-KK collected part of the data, cleaned and analyzed the data, and drafted the manuscript. OH and BA analyzed the data and drafted the manuscript. M-LC drafted the manuscript. HC conceived the study and drafted the manuscript. All authors contributed to the article and approved the submitted version.
