# Supplementary material for: Self-management training vs. neurofeedback interventions for attention deficit hyperactivity disorder: Results of a randomized controlled treatment study
Source: Front Psychiatry. 2022 Aug 18;13:969351. doi: 10.3389/fpsyt.2022.969351 (PMC9433654; doi:10.3389/fpsyt.2022.969351)
Supplement: Supplementary file 1 [file Data_Sheet_1.PDF]

Psychological interventions for ADHD: Results of a randomized controlled treatment study

Supplement

Authors: Ann-Kathrin Korfmacher [a,*], Oliver Hirsch [b], Mira-Lynn Chavanon [a], Björn Albrecht [a], Hanna Christiansen [a]

Affiliations:

[a] Clinical Child and Adolescent Psychology, Department of Psychology, Philipps-University Marburg, Gutenbergstraße 18, 35032 Marburg, Germany

[b] Department of Psychology, FOM University of Applied Sciences, Birlenbacher Str. 17, 57078 Siegen, Germany

*corresponding author

E-Mail: ann-kathrin.korfmacher@uni-marburg.de

Per Protocol Analyses

Treatment Effects from Baseline (T1) to FollowUp (T3) per protocol

Analyses limited to the patients that completed their interventions per protocol, n = 49 receiving SMT and n = 46 NF, generally confirmed the mITT findings with the exception that SMT was found to be superior to NF in the self-concept interview Δ School, which was not the case in the mITT analysis (Supplementary Table S1; PP: $F_{(1, 93)} = 3.7$, $p = .06$, part. $\eta^2 = .04$ vs. mITT: $F_{(1, 111)} = 2.3$, $p = .13$, part. $\eta^2 = .02$).

Limiting the analysis to the PP-sample that finished their allocated intervention without imputing missing data was generally in line with that, except that also the Conners' parent-rated Δ Hyperactivity/Impulsivity (DSM) indicated superiority of SMT over NF (Supplementary Table S2; PP: $F_{(1, 61)} = 5.4$, $p = .02$, part. $\eta^2 = .08$ vs. mITT: $F_{(1, 111)} = 0.9$, $p = .34$, part. $\eta^2 < .01$).

Supplementary Table S1: Change of the main Outcome Criteria from Baseline (T1) to Post-Treatment (T3, per protocol, after imputing missing values with k nearest neighbor (kNN) algorithm)

	Self-Management Training n=49	Neurofeedback n=46	
**Primary outcome**			
Conners parent-rated[1]			
Δ ADHD-Index	**-7.1* (8.1)**	**-4.8* (8.0)**	$F_{(1, 93)}$=1.8, p=.17, part. η^2=.02
Δ Hyperactivity/Impulsivity (DSM)	**-7.9* (8.8)**	**-6.6* (9.7)**	$F_{(1, 93)}$=0.5, p=.50, part. η^2<=.01
Δ Inattention (DSM)	**-5.5* (8.0)**	**-3.6* (8.7)**	$F_{(1, 93)}$=1.3, p=.26, part. η^2=.01
Conners teacher-rated[2]			
Δ ADHD-Index	2.6 (11.0)	2.0 (7.4)	$F_{(1, 93)}$=0.1, p=.74, part. η^2<=.01
Δ Hyperactivity/Impulsivity (DSM)	**-4.5* (10.8)**	-2.4 (8.5)	$F_{(1, 93)}$=1.1, p=.29, part. η^2=.01
Δ Inattention (DSM)	0.7 (9.5)	1.0 (7.7)	$F_{(1, 93)}$=0.0, p=.87, part. η^2<=.01
**Secondary outcome**			
Responder-Rate[3]			
Qb-Test[4]			
Δ Hyperactivity	**-0.4* (1.4)**	-0.1 (1.4)	$F_{(1, 93)}$=1.3, p=.26, part. η^2=.01
Δ Impulsivity	**-0.4* (1.2)**	**-0.5* (0.9)**	$F_{(1, 93)}$=0.2, p=.66, part. η^2<=.01
Δ Inattention	**-0.5* (1.2)**	0.1 (1.1)	**$F_{(1, 93)}$=5.4, p=.02, part. η^2=.06**
KINDL[5]			
Δ Self-Esteem	0.3 (1.2)	0.1 (1.0)	$F_{(1, 93)}$=0.7, p=.41, part. η^2<=.01
Δ School	0.3 (0.9)	-0.3 (1.1)	**$F_{(1, 93)}$=7.2, p<.01, part. η^2=.07**
Δ Family	0.2 (0.8)	0.2 (0.9)	$F_{(1, 93)}$=0.0, p=.97, part. η^2<=.01
Δ Peers	0.2 (1.1)	-0.1 (1.1)	$F_{(1, 93)}$=2.0, p=.16, part. η^2=.02
Self-Concept Interview[6]			
Δ School	**0.3* (0.7)**	0.1 (0.7)	**$F_{(1, 93)}$=3.7, p=.06, part. η^2=.04**
Δ Family	**0.2* (0.5)**	0.1 (0.6)	$F_{(1, 93)}$=1.4, p=.25, part. η^2=.01
Δ Peers	**0.2* (0.5)**	0.1 (0.7)	$F_{(1, 93)}$=1.0, p=.33, part. η^2=.01

* significant <u>improvements</u> from T1 to T3 (p<.05, two-tailed)

[1] missing at T1 for N=5 children receiving SMT (n=2) or NF (n=3) and at Post (T3) for additional N=27 children receiving SMT (n=13) or NF (n=14), imputed.

[2] missing at T1 for N=8 children receiving SMT (n=2) or NF (n=6) and at Post (T3) for additional N=29 children receiving SMT (n=17) or NF (n=12), imputed.

[3] more than 20% reduction from T1 to T2 in Conners parent-rated ADHD-index; missing at T1 or T3 for N=32 children receiving SMT (n=15) or NF (n=17), imputed.

[4] missing at T1 and/or T3 for N=26 children receiving SMT (n=13) or NF (n=13), imputed.

[5] missing at T1 for N=7 children receiving SMT (n=3) or NF (n=4) and at Post (T3) for additional N=7 children receiving SMT (n=4) or NF (n=3), imputed.

[6] missing at T1 for N=3 children receiving SMT (n=1) or NF (n=2) and at Post (T3) for additional N=3 children receiving SMT (n=2) or NF (n=1), imputed.

Supplementary Table S2: Change of the main Outcome Criteria from Baseline (T1) to Post-Treatment (T3, per protocol, without imputations)

	Self-Management Training n=49	Neurofeedback n=46	
**Primary outcome**			
Conners parent-rated[1]			
Δ ADHD-Index	**-7.9* (8.8)**	**-6.3* (7.6)**	$F_{(1, 61)}$=0.5, p=.47, part. η^2<=.01
Δ Hyperactivity/Impulsivity (DSM)	**-9.4* (8.7)**	**-4.0* (9.4)**	**$F_{(1, 61)}$=5.4, p=.02, part. η^2=.08**
Δ Inattention (DSM)	**-5.9* (8.6)**	**-5.6* (9.0)**	$F_{(1, 61)}$=0.0, p=.88, part. η^2<=.01
Conners teacher-rated[2]			
Δ ADHD-Index	2.1 (11.4)	0.8 (8.6)	$F_{(1, 56)}$=0.2, p=.64, part. η^2<=.01
Δ Hyperactivity/Impulsivity (DSM)	**-7.7* (11.6)**	-4.3 (9.1)	$F_{(1, 56)}$=1.6, p=.22, part. η^2=.03
Δ Inattention (DSM)	-0.2 (10.3)	-1.4 (7.6)	$F_{(1, 56)}$=0.2, p=.64, part. η^2<=.01
**Secondary outcome**			
Responder-Rate[3]	5/34 (14.7%)	7/29 (24.1%)	$X^2_{(1)}$=0.90, p=.34
Qb-Test[4]			
Δ Hyperactivity	-0.4 (1.5)	0.1 (1.5)	$F_{(1, 67)}$=1.7, p=.19, part. η^2=.03
Δ Impulsivity	-0.5 (1.2)	-0.5 (0.8)	$F_{(1, 67)}$=0.0, p=.99, part. η^2<.01
Δ Inattention	-0.4 (1.2)	0.1 (1.0)	**$F_{(1, 67)}$=4.6, p=.04, part. η^2=.06**
KINDL[5]			
Δ Self-Esteem	0.2 (1.2)	0.1 (1.0)	$F_{(1, 79)}$=0.0, p=.91, part. η^2<=.01
Δ School	0.2 (0.9)	-0.3 (1.0)	**$F_{(1, 79)}$=4.5, p=.04, part. η^2=.05**
Δ Family	0.1 (0.8)	0.1 (0.9)	$F_{(1, 79)}$=0.0, p=.96, part. η^2<=.01
Δ Peers	0.2 (1.1)	-0.1 (1.2)	$F_{(1, 79)}$=1.8, p=.18, part. η^2=.02
Self-Concept Interview[6]			
Δ School	**0.3* (0.7)**	0.0 (0.7)	**$F_{(1, 87)}$=2.9, p=.09, part. η^2=.03**
Δ Family	**0.2* (0.5)**	0.1 (0.6)	$F_{(1, 87)}$=0.2, p=.62, part. η^2<=.01
Δ Peers	**0.2* (0.5)**	0.1 (0.7)	$F_{(1, 87)}$=0.8, p=.37, part. η^2<=.01

[*] significant <u>improvements</u> from T1 to T3 (p<.05, two-tailed)

[1] missing at T1 for N=5 children receiving SMT (n=2) or NF (n=3) and at Post (T3) for additional N=27 children receiving SMT (n=13) or NF (n=14), thus df=1, 61.

[2] missing at T1 for N=8 children receiving SMT (n=2) or NF (n=6) and at Post (T3) for additional N=29 children receiving SMT (n=17) or NF (n=12), thus df=1, 56.

[3] more than 20% reduction from T1 to T2 in Conners parent-rated ADHD-index; missing at T1 or T3 for N=32 children receiving SMT (n=15) or NF (n=17), thus df=1, 67.

[4] missing at T1 and/or T3 for N=26 children receiving SMT (n=13) or NF (n=13), thus df=1, 67.

[5] missing at T1 for N=7 children receiving SMT (n=3) or NF (n=4) and at Post (T3) for additional N=7 children receiving SMT (n=4) or NF (n=3), thus df=1, 79.

[6] missing at T1 for N=3 children receiving SMT (n=1) or NF (n=2) and at Post (T3) for additional N=3 children receiving SMT (n=2) or NF (n=1), thus df=1, 87.

Supplementary Table S3: Original path coefficients of Conners subscales in parents and teachers in NF (n = 44) and SMT (n = 48) after imputing missing values with k nearest neighbor (kNN) algorithm

Scale	Path Coefficients Original (NF)	Path Coefficients Original (SMT)	p value difference	Path Coefficients Bootstrap (NF)	Path Coefficients Bootstrap (SMT)
Imputed	n = 44	n = 48			
Conners Hyperactivity Parents	.746	.614	.16	.772 (p < .001)	.659 (p < .001)
Conners Inattention Parents	.658	.452	.51	.692 (p < .001)	.529 (p = .134)
Conners Impulsivity Parents	.704	.701	.98	.714 (p < .001)	.709 (p < .001)
Not Imputed	n=39	n=45			
Conners Hyperactivity Teachers	.698	.654	.67	.719 (p < .001)	.682 (p < .001)
Conners Inattention Teachers	.377	.540	.69	.374 (p = .35)	.594 (p < .001)
Conners Impulsivity Teachers	.674	.612	.64	.689 (p < .001)	.623 (p < .001)

Note: Original path coefficients of Conners subscales in parents and teachers in Neurofeedback (NF) and Self-management (SMT) groups, the p value of the Welch-test of differences and the mean path coefficients in both groups after 5000 bootstrap samples and their significance values.

Supplementary Table S4: Original path coefficients of Qb-Test subscales based on Q values of Qb+ variables in NF (n = 33) and SMT (n = 36) after imputing missing values with k nearest neighbor (kNN) algorithm.

Scale	Path Coefficients Original (NF)	Path Coefficients Original (SMT)	p value difference	Path Coefficients Bootstrap (NF)	Path Coefficients Bootstrap (SMT)
Qb-Test Hyperactivity	.366	.393	.92	.403 (p = .045)	.414 (p = .036)
Qb-Test Inattention	.723	.562	.31	.738 (p < .001)	.619 (p < .001)
Qb-Test Impulsivity	.564	.390	.68	.600 (p < .001)	.250 (p = .34)

Note: Original path coefficients of Qb-Test subscales in Neurofeedback (NF) and Self-management (SMT) groups, the p value of the Welch-test of differences and the mean path coefficients in both groups after 5000 bootstrap samples and their significance values.

Supplementary Table S5: Original path coefficients of KINDL subscales in NF (n = 43) and SMT (n = 46) after imputation of missing values with k nearest neighbor (kNN) algorithm.

Scale	Path Coefficients Original (NF)	Path Coefficients Original (SMT)	p value difference	Path Coefficients Bootstrap (NF)	Path Coefficients Bootstrap (SMT)
KINDL Physical	.558	.471	.70	.603 (p < .001)	.524 (p = .015)
KINDL Self-Esteem	.594	.444	.51	.627 (p < .001)	.481 (p = .02)
KINDL Family	.541	.382	.58	.577 (p = .024)	.509 (p = .012)
KINDL Peer	.516	.566	.87	.530 (p = .067)	.618 (p < .001)
KINDL School	.383	.482	.74	.491 (p = .073)	.532 (p = .019)
KINDL Emotional	.497	.570	.89	.507 (p = .12)	.442 (p = .18)

Note: Original path coefficients of KINDL subscales in Neurofeedback (NF) and Self-management (SMT) groups, the p value of the Welch-test of differences and the mean path coefficients in both groups after 5000 bootstrap samples and their significance values.

Supplementary Table S6: Original path coefficients of self-concept interview subscales in NF (n = 43) and SMT (n = 46) after imputation of missing values with k nearest neighbor (kNN) algorithm.

Scale	Path Coefficients Original (NF)	Path Coefficients Original (SMT)	p value difference	Path Coefficients Bootstrap (NF)	Path Coefficients Bootstrap (SMT)
SC Body	.568	.712	.36	.631 ($p < .001$)	.743 ($p < .001$)
SC Family	.614	.675	.64	.718 ($p < .001$)	.735 ($p < .001$)
SC Peers	.651	.662	.96	.691 ($p = .001$)	.712 ($p < .001$)
SC School	.535	.508	.89	.631 ($p < .001$)	.621 ($p < .001$)

Note: Original path coefficients of self-concept interview subscales in Neurofeedback (NF) and Self-management (SMT) groups, the p value of the Welch-test of differences and the mean path coefficients in both groups after 5000 bootstrap samples and their significance values.

Supplementary Table S7a: The internal consistency of latent constructs at Baseline (T1, mITT) in SMT (n = 58) and NF (n = 55)

	Self-Management Training n = 58				Neurofeedback n = 55			
Primary outcome	Cronbach´s Alpha	Rho_A	Composite Reliability	Average Variance Extracted (AVE)	Cronbach´s Alpha	Rho_A	Composite Reliability	Average Variance Extracted (AVE)
Conners Parents Hyperactivity	0.873	0.890	0.897	0.453	0.879	0.892	0.900	0.454
Conners Parents Impulsivity	0.809	0.853	0.887	0.725	0.742	0.757	0.854	0.662
Conners Parents Inattention	0.784	0.768	0.824	0.331	0.856	0.894	0.884	0.441
Conners Teacher Hyperactivity	0.921	0.927	0.933	0.503	0.958	0.963	0.963	0.649
Conners Teacher Impulsivity	0.872	0.895	0.912	0.722	0.923	0.940	0.946	0.813
Conners Teacher Inattention	0.892	0.917	0.915	0.532	0.880	0.887	0.903	0.524

Supplementary Table S7b: The internal consistency of latent constructs at Baseline (T1, mITT) in SMT (n = 58) and NF (n = 55)

	Self-Management Training n = 58				Neurofeedback n = 55			
Secondary outcome	Cronbach´s Alpha	Rho_A	Composite Reliability	Average Variance Extracted (AVE)	Cronbach´s Alpha	Rho_A	Composite Reliability	Average Variance Extracted (AVE)
Qb Hyperactivity	0.929	0.984	0.948	0.799	0.936	0.989	0.955	0.815
Qb Impulsivity	0.789	-1.755	0.630	0.422	0.702	0.711	0.833	0.626
Qb Inattention	0.769	0.881	0.858	0.672	0.719	0.735	0.840	0.639
KINDL Emotional	0.383	0.799	0.426	0.271	0.267	0.632	0.201	0.415
KINDL Family	0.642	0.720	0.777	0.485	0.618	0.721	0.743	0.445
KINDL Peer	0.677	0.739	0.753	0.481	0.560	0.496	0.628	0.423
KINDL Physical	0.510	0.611	0.716	0.409	0.541	0.661	0.726	0.421
KINDL School	0.358	0.817	0.639	0.409	0.362	0.376	0.689	0.383
KINDL self-esteem	0.763	0.879	0.839	0.571	0.781	0.819	0.857	0.602
SK Body	0.464	0.466	0.698	0.372	0.449	0.669	0.685	0.379
SK Family	0.870	0.883	0.889	0.357	0.763	0.796	0.798	0.241
SK Peers	0.737	0.757	0.811	0.357	0.702	0.640	0.686	0.252
SK School	0.785	0.821	0.842	0.410	0.776	0.863	0.824	0.386

Supplementary Table S8a: The internal consistency of latent constructs at Post-Treatment (T3, mITT) in SMT (n = 58) and NF (n = 55)

	Self-Management Training n = 58				Neurofeedback n = 55			
Primary outcome	Cronbach´s Alpha	Rho_A	Composite Reliability	Average Variance Extracted (AVE)	Cronbach´s Alpha	Rho_A	Composite Reliability	Average Variance Extracted (AVE)
Conners Parents Hyperactivity	0.863	0.883	0.889	0.428	0.888	0.897	0.908	0.478
Conners Parents Impulsivity	0.894	0.896	0.934	0.825	0.772	0.773	0.868	0.687
Conners Parents Inattention	0.861	0.896	0.893	0.476	0.886	0.916	0.909	0.516
Conners Teacher Hyperactivity	0.943	0.951	0.950	0.581	0.944	0.953	0.952	0.590
Conners Teacher Impulsivity	0.881	0.890	0.919	0.739	0.910	0.919	0.937	0.788
Conners Teacher Inattention	0.894	0.914	0.915	0.529	0.901	0.930	0.921	0.552

Supplementary Table S8b: The internal consistency of latent constructs at Post-Treatment (T3, mITT) in SMT (n = 58) and NF (n = 55)

	Self-Management Training n = 58				Neurofeedback n = 55			
Secondary outcome	Cronbach´s Alpha	Rho_A	Composite Reliability	Average Variance Extracted (AVE)	Cronbach´s Alpha	Rho_A	Composite Reliability	Average Variance Extracted (AVE)
Qb Hyperactivity	0.942	0.977	0.959	0.829	0.968	1.000	0.976	0.890
Qb Impulsivity	0.755	0.602	0.730	0.514	0.806	0.935	0.877	0.705
Qb Inattention	0.569	0.660	0.739	0.502	0.764	0.795	0.860	0.673
KINDL Emotional	0.539	0.317	0.450	0.349	0.213	-0.214	0.005	0.222
KINDL Family	0.534	0.574	0.726	0.406	0.585	0.513	0.712	0.407
KINDL Peer	0.634	0.717	0.747	0.503	0.761	0.816	0.850	0.592
KINDL Physical	0.697	0.878	0.785	0.499	0.740	0.780	0.839	0.571
KINDL School	0.446	0.627	0.695	0.406	0.600	0.644	0.767	0.460
KINDL self-esteem	0.784	0.806	0.847	0.588	0.857	0.888	0.903	0.701
SK Body	0.486	0.547	0.708	0.398	0.548	0.750	0.717	0.409
SK Family	0.846	0.868	0.872	0.325	0.852	0.889	0.875	0.342
SK Peers	0.730	0.805	0.772	0.358	0.830	0.871	0.867	0.458
SK School	0.763	0.808	0.806	0.351	0.789	0.846	0.829	0.407
